# OP63 A European Value Assessment Framework For Next-Generation Sequencing And Comprehensive Genomic Profiling Oncology Diagnostics

**DOI:** 10.1017/S0266462324001235

**Published:** 2025-01-07

**Authors:** Federico Augustovski, Danitza Chavez, Madeleine Haig, Caitlin Main, Fernando Argento, Carla Colaci, Mackenzie Mills, Verónica Alfie, Andres Pichon-Riviere, Andrea Alcaraz, Panos Kanavos

## Abstract

**Introduction:**

Advances in next-generation sequencing/comprehensive genomic profiling (NGS/CGP) diagnostics require a value framework (VF) for accurate assessment within cancer care in Europe. Building on a previously established VF for diagnostic technologies in Latin America by the Institute for Clinical Effectiveness and Health Policy (IECS), this study synthesizes insights from a systematic literature review and stakeholder perspectives to inform the co-creation of an NGS/CGP diagnostic framework for healthcare decision-making.

**Methods:**

The study utilized a mixed methods approach including a systematic literature review (SLR) and web-Delphi panel. The SLR identified and mapped existing VFs against the IECS VF, extracting non-overlapping criteria relevant to NGS/CGP in Europe. A Delphi panel further adapted and validated the framework, ensuring comprehensive stakeholder contribution and engagement. The Delphi´s first round was qualitative and involved open-ended feedback from participants to adapt the indicators and domains. Rounds two through four involved Likert-scale judgments of importance of each indicator. In rounds three and four, participants were shown the distribution of responses across stakeholders and could reconsider their answers.

**Results:**

The SLR revealed 42 VFs with an 83 percent criterion overlap with the IECS VF, resulting in 46 indicators forming the literature-adapted framework. Thirty-four participants completed the Delphi. In round one, 14 indicators and 22 descriptions were adapted, 11 indicators were merged, 14 were deleted, and one was kept the same resulting in 29 indicators for scoring in round two. The final VF has 27 indicators: 23 essential and four complementary; two indicators did not reach consensus. The domains included clinical impact, test performance, scientific evidence quality, non-clinical impact, health system integration, economic aspects, ethical/governance concerns, and health system priorities.

**Conclusions:**

This approach has yielded a robust, stakeholder co-created VF for NGS/CGP diagnostics in oncology, tailored to the European setting. It offers a comprehensive set of criteria that extends beyond traditional health technology assessments, incorporating novel aspects like post-test data governance. This framework sets the stage for improving patient access to high-value technologies by aligning European stakeholder values across health systems.

